# Prion‐like mechanisms in neurodegenerative disease: Implications for Huntington's disease therapy

**DOI:** 10.1002/sctm.19-0248

**Published:** 2020-01-30

**Authors:** Bhairavi Srinageshwar, Robert B. Petersen, Gary L. Dunbar, Julien Rossignol

**Affiliations:** ^1^ College of Medicine Central Michigan University Mount Pleasant Michigan; ^2^ Field Neurosciences Institute Laboratory for Restorative Neurology Central Michigan University Mount Pleasant Michigan; ^3^ Program in Neuroscience Central Michigan University Mount Pleasant Michigan; ^4^ Department of Psychology Central Michigan University Mount Pleasant Michigan; ^5^ Field Neurosciences Institute Saginaw Michigan

**Keywords:** Huntington's disease, mHTT aggregates, prion, stem cells, transplantation

## Abstract

Huntington's disease (HD) is an autosomal dominant neurodegenerative disorder caused by a CAG repeat expansions in the huntingtin gene resulting in the synthesis of a misfolded form of the huntingtin protein (mHTT) which is toxic. The current treatments for HD are only palliative. Some of the potential therapies for HD include gene therapy (using antisense oligonucleotides and clustered regularly interspaced short palindromic repeats‐Cas9 system) and stem‐cell‐based therapies. Various types of stem cell transplants, such as mesenchymal stem cells, neural stem cells, and reprogrammed stem cells, have the potential to either replace the lost neurons or support the existing neurons by releasing trophic factors. Most of the transplants are xenografts and allografts; however, recent reports on HD patients who received grafts suggest that the mHTT aggregates are transferred from the host neurons to the grafted cells as well as to the surrounding areas of the graft by a “prion‐like” mechanism. This observation seems to be true for autotransplantation paradigms, as well. This article reviews the different types of stem cells that have been transplanted into HD patients and their therapeutic efficacy, focusing on the transfer of mHTT from the host cells to the graft. Autotransplants of reprogramed stem cells in HD patients are a promising therapeutic option. However, this needs further attention to ensure a better understanding of the transfer of mHTT aggregates following transplantation of the gene‐corrected cells back into the patient.

## Graphical abstract

The HD brains have neurons with mutant form of the gene leading to the formation of aggregates. Stem‐cell‐based therapies have shown great promise; however, there is a risk of transferring the aggregates from the host cells to the transplanted stem cells. There is an open question as to whether the transfer takes place in a “prion‐like” fashion.

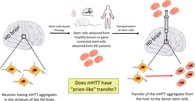

**Significance statement**

Stem cell transplantation, along with gene editing using a variety of molecular tools, is one of the most promising strategies that is being investigated by many researchers as a potential treatment for neurodegenerative diseases. Huntington's disease (HD) is one of the neurodegenerative diseases in which transplantation has been widely studied using different types of innate as well as reprogrammed/modified stem cells as a potential therapy. Transplantation using different types of stem cells, such as mesenchymal stem cells, neural stem cells, embryonic stem cells, and induced pluripotent stem cells accompanied by clustered regularly interspaced short palindromic repeats‐Cas9‐based gene editing, was performed in laboratory settings, which could have an impact in the clinics in the near future. Though the treatment strategies had encouraging outcomes, one of the major issues identified recently was that mutant huntingtin protein aggregates transfer from the HD cells to the wild‐type/transplanted cells in the host brain, by a “prion‐like” mechanism. The finding brings into question to what extent these stem cell/gene‐corrected cell transplants are a viable option for treating HD.

## 1 INTRODUCTION

Stem cell therapy has the potential to make a major impact on the treatment of many diseases, especially neurodegenerative diseases. Stem‐cell‐based therapies have shown great promise in animal models of Huntington's (HD), Parkinson's (PD), and Alzheimer's (AD) diseases which has led to clinical trials that are at various stages of completion.^1^ The most widely used stem cells are mesenchymal stem cells (MSCs), which are derived from a variety of sources including bone‐marrow derived mesenchymal stem cells (BM‐MSCs), umbilical cord blood‐derived mesenchymal stem cells (UC‐MSCs), Wharton jelly‐derived mesenchymal stem cells, induced pluripotent stem cells (iPSCs), neural stem cells (NSCs), and embryonic stem cells (ESCs).^2^

Given the importance of developing new therapies to counteract neurodegenerative processes and the enormous potential of using stem cells for treating neurodegenerative diseases, this concise review focuses on the new avenues for exploring how stem cells, per se, and especially how reprogramming stem cells play pivotal roles in the development of effective stem‐cell‐based therapies for treating HD, a particularly devastating neurodegenerative disorder.

## 2 HUNTINGTON'S DISEASE

Huntington's disease (HD) is an autosomal dominant, late‐onset, neurodegenerative disorder, which primarily affects the caudate and putamen regions of the brain. The medium spiny GABAergic neurons (MSNs) are the most affected cell type. The disease is linked to a triplet repeat expansion, CAG, in the N‐terminal coding region of the Huntingtin (*HTT*) gene leading to the production of an abnormal, misfolded mutant form of the huntingtin protein (mutant HTT or mHTT) that aggregates in the nucleus and cytoplasm of neurons.^3^ The mutation causes a gain of function, whereby the toxic effects of mHTT leads to neuronal degeneration. The presence of mHTT also interferes with protein synthesis and quality control (proteostasis) leading to impaired cellular function and loss of viability. There is currently no cure or effective treatment for HD.^4^, ^5^

The triad of symptoms found in HD includes (a) motor symptoms, commonly known as Huntington's chorea; (b) cognitive decline; and (c) psychiatric disturbances and mood disorders.^6^ In addition to the presence of aggregated mHTT, HD is also associated with high levels of neuroinflammation.^7^

## 3 CLINICAL STUDIES USING STEM CELL TRANSPLANTS IN HD PATIENTS

There are many clinical studies in which stem cells were transplanted into HD patients; selected studies are discussed below.

Human fetal NSCs were transplanted into the caudate, pre‐commissural putamen, and post‐commissural putamen in five patients who were diagnosed with HD. Follow‐up on patients showed that the transplanted cells were detectable in the brain even 1 year after transplantation. The motor deficits in the patients were ameliorated to some extent starting at 3 months post‐surgery as defined by the Unified Huntington's Disease Rating Scale (UHDRS). However, a few of the pitfalls of this study were (i) atrophy at the site of transplantation, (ii) negative effects of immunosuppressants, and (iii) stress in patients that contributed to difficulties at the long‐term follow‐up visit.^8^

In another study, fetal stem cells were transplanted into seven symptomatic HD patients using bilateral intracranial injections. Following transplantation, the patients were evaluated using the UHDRS and a behavior analysis over the course of 12 months. The study found a trend in improvement in patients' motor function following transplantation; however, the symptoms did not worsen in patients. The literature provides evidence that HD patients have a decrease in striatal metabolic glucose and dopamine binding efficacy. This study shows that there was no such decrease in glucose metabolism or dopamine binding efficacy in HD patients following treatment, demonstrating the potential treatment effect of transplanting fetal stem cells.^9^

Yet another study transplanted human fetal stem cells unilaterally into HD patients by stereotactic guided intracranial injection into the caudate and putamen. The patients were immunosuppressed following transplantation and assessed using MRI and clinical scoring to study the potential effects of the transplants. MRI scans showed the presence of transplanted cells/grafts in the striatum of the HD patients. The motor and cognitive deficits were monitored in these patients based on the UHDRS from 6 to 60 months. Although the data did not show a significant treatment effect, a trend toward improvement was seen.^10^

A recent study from 2018 analyzed the first clinicopathological outcome following a single bilateral transplantation of a fetal striatal allograft into an HD patient. Though the patient did not show any improvement following transplantation, one of the striking histological observations is that mHTT aggregates were present in the grafted tissue, showing that the mHTT was transferred from the host cells to the graft cells.^11^

## 4 STEM‐CELL‐BASED THERAPY IN HD RODENT MODELS

### 4.1 Use of iPSCs as a therapy for HD

iPSCs are pluripotent stem cells that are derived from the patient's own skin cells, fibroblasts, or many other sources. Once the cells are induced to become pluripotent they can be reprogrammed into a variety of cell types, including neuronal lineages, which can then be transplanted to correct different disease conditions.^12^

Mu and colleagues (2014) performed ventricular transplantation of iPSCs into the striata of rats given quinolinic acid (QA), mimicking HD neuropathology. Analysis of brain tissue showed that the iPSCs, following transplantation, migrated to the lesion site and rendered a therapeutic effect. The HD rats exhibited improved cognitive function as assessed by the Morris water maze test. Moreover, partial protection from QA‐induced atrophy (ie, loss of striatal volume) following transplantation was observed. The transplanted iPSCs differentiated into medium spiny neurons and glial cells as determined by histological analysis and cell counts.^13^

In a recent study, we performed intrastriatal transplantation of induced neural stem cells (iNSCs), obtained from iPSCs, into the striatum of YAC128 mice, that carry the full‐length human HD gene. We found that the iNSCs differentiated into medium spiny neurons, resulting in significantly higher cell counts in the transplanted mice compared with the vehicle‐treated mice. This, in turn, reduced the motor symptoms observed in the transplanted YAC128 mice. As it is well known, brain‐derived neurotrophic factor (BDNF) is known to be reduced in the HD brain, which has critical implications for neuronal survival. Our study showed that following iNSCs transplantation, the BDNF protein and its receptor, Trkβ, were significantly higher in the transplanted group.^14^

In another study, MSNs derived from human pluripotent stem cells were packaged in a three‐dimensional hydrogel which was then transplanted bilaterally into the striatum of R6/2 mice. The mice were tested using a battery of behavioral tests for 7 weeks post‐transplantation. The study results showed that the mice had reduced motor deficits as evidenced by reduced clasping and increased latency to fall from the rotarod. Moreover, the transplanted HD mice lived beyond their defined life span (∼19% increase) compared with the untreated, control mice.^15^ However, the transplanted cells in the HD brain contained mHTT aggregates in the nucleus, proving that the aggregates were transferred from the host cells to the transplanted cells.

Importantly, no tumors were reported in the iPSC studies described here, indicating that iPSC‐based therapy is a safe and feasible treatment option for HD.

### 4.2 Use of MSCs as a therapy for HD

MSCs are the most widely used stem cells for treating neurodegenerative disorders.^16^ Transplantation of MSCs into different transgenic mouse models of HD, for example, R6/2, YAC128, BACHD, as well as the QA‐ and 3‐nitropropionic acid‐rat models of HD has provided evidence that MSC transplants can reduce HD‐like deficits.^17^, ^18^, ^19^, ^20^ MSCs have anti‐inflammatory properties and secrete trophic factors that promote the survival of neurons. Many studies conducted in our laboratory focused on the use of BM‐MSCs as a potential therapy for HD following their transplantation into different mouse lines carrying mutant forms of the *HTT* gene.^17^, ^20^

Dey and colleagues (2010) found that intrastriatal transplantation of MSCs genetically altered to overexpress BDNF, which is reduced in HD patients,^21^ decreased the motor deficits and neuronal loss in YAC128 HD mice.^20^ We also transplanted MSCs derived from UC‐MSCs into the striata of R6/2 HD mice. We found that UC‐MSC transplants reduced learning and memory deficits but did not improve motor symptoms in these mice.^22^ These studies suggest that the source of MSCs may produce different outcomes. A 2018 study transplanted human umbilical cord matrix stem cells into the striata of 3‐NP rats, and the tissue was analyzed 1 month following transplantation. The study showed that the transplanted stem cells survived and increased dendritic spine length as well as the volume of the striatum in the brain. Behavioral analysis showed that the HD rats regained their motor activity demonstrating the therapeutic effects of the transplanted cells. Prior to transplantation, the cells were shown to secrete glial cell‐derived neurotrophic factor and vascular endothelial growth factor.^23^

In addition to the administration of MSCs via invasive intracranial injections, a 2019 study administered BM‐MSCs using an intranasal route in R6/2 HD mice. The MSCs reached the brain, and a battery of behavioral tests showed that the MSCs exerted therapeutic effects, reducing the motor deficits and symptoms. Moreover, the MSC‐treated mice survived longer than their control littermates. The treated mice also showed a regular sleep cycle pattern and normal circadian rhythms compared with the untreated mice. This study also analyzed the dopamine signaling cascade by determining the tyrosine hydroxylase and dopamine‐regulated neuronal phosphoprotein (DARPP‐32) levels and found that MSCs rescued the deficits in the cascade to some extent.^24^

A very recent study used a calcium channel blocker, lercanidipine (LER), in addition to BM‐MSCs transplantation in 3‐NP HD rats. Behavioral analysis showed that the combinatorial therapy improved the behavioral as well as motor deficits as evidenced by improvements in open field and grip strength tasks. Moreover, BM‐MSCs themselves have anti‐inflammatory properties, and the combination with LER was found to be more effective in reducing inflammation compared with MSCs alone. The study also found that LER treatment reduced cytosolic calcium levels and modulated some signaling pathways, for example, Wnt and β‐catenin. These changes were notably increased following combination therapy with LER and BM‐MSCs.^25^

Two of us (G.L.D. and J.R., 2015) found that transplantation of BM‐MSCs at lower passage numbers (P3‐P8) secreted less BDNF and were less effective in reducing motor deficits than higher passaged (P40‐P50) BM‐MSCs in R6/2 mice.^17^ Thus, both the source of the MSCs and the number of times they were passaged can have a significant effect on their therapeutic efficacy.

Another study found that genetically manipulated human‐derived MSCs that overexpress BDNF are therapeutically effective following their transplantation into immune‐suppressed YAC128 and R6/2 HD mice. Transplantation of these cells reduced measures of anxiety and increased endogenous neurogenesis.^26^ In addition, Snyder and colleagues (2010) showed that transplantation of bone marrow‐derived human multipotent stromal stem cells into the dentate gyrus of transgenic N171‐82Q HD mice increased the proliferative and differentiation capacity of the endogenous neural progenitor cells. This, in turn, led to increased trophic support and neurogenesis in the striatum of these transplanted mice.^27^ Transplantation of adipose tissue‐derived stem cells (ASCs) into rats given intrastriatal injections of QA reduced both motor symptoms and lesion volume. In addition, these investigators found that ASCs alleviated motor deficits and increased neurogenesis in 8‐week‐old R6/2 mice. Furthermore, the number of mHTT aggregates was significantly reduced, and the median life span of these mice was increased.^28^ Collectively, these studies indicate that MSC transplants confer significant therapeutic effects in a variety of HD rodent models, but variables such as MSC source and number of passages need to be to be optimized to achieve the best neuroprotective effects.

### 4.3 Use of NSCs as therapy for HD

NSCs are another promising type of stem cell currently being used as a potential stem‐cell‐based therapy for HD. A recent report by Reidling and colleagues (2018) showed that transplantation of human embryonic stem‐cell‐derived neural stem cells (hNSCs) into the striatum of R6/2 HD mice reduced motor deficits and synaptic alterations. Moreover, these transplanted stem cells were integrated into the appropriate endogenous neural circuit, as demonstrated by electrophysiological studies.^29^ Studies assessing the efficacy of transplanting hNSCs into the striata of 3‐NP‐treated rats demonstrated sparing of both motor deficits and striatal neurons as a result of increased BDNF production in the brains of those rats.^30^

### 4.4 Autologous and induced pluripotent cells as a therapy for HD

Given the different types of stem cell transplants used to treat HD, transplantation of autologous stem cells is of special interest because this type of transplant uses the patient's own cells, avoiding the need for immunosuppression. We have studied autologous transplantation of BM‐MSCs into rats following intrastriatal QA injections to assess therapeutic outcomes from transplantation of these cells.^31^ Positive outcomes of autologous transplants in chemically induced models of HD can provide a general idea of feasibility, but testing with genetic HD models is required to assess the potential clinical efficacy of transplants of either autologous stem cells or gene‐edited autologous cells. An and colleagues (2012) corrected iPSCs derived from HD patients, using homologous recombination, and showed that correction of the iPSCs did not alter the innate characteristics of the stem cells. NSCs were derived from these corrected iPSCs and then transplanted into R6/2 HD mice. Histological analysis showed that the corrected transplanted cells survived and were able to differentiate into region specific cells, thereby repopulating the striatal area with the appropriate cell types.^32^

Table 1 summarizes the study outcomes and transfer of HTT aggregates from host cells to donor cells following stem cell transplantations in transgenic HD mice as well as in some clinical trials.

**Table 1:** Report of HD aggregates discussions after stem cell transplantations in transgenic HD models and selected clinical trials

Study	HD model	Stem cells transplanted	Source of the transplanted cells	Study outcomes	Aggregates in transplanted cells
Lee et al^28^	R6/2 mice	Adipose tissue derived stem cells (ASCs)	Healthy human donors	Reduction in mHTT aggregates and lesser motor deficits with improved survival was observed in R6/2 mice	Not discussed
Snyder et al^27^	N171‐82Q transgenic mice	Multipotent stromal cells from bone marrow	Healthy human donors	The transplanted cells increased endogenous neural stem cell proliferation thereby increasing the trophic support, decreasing atrophy and increasing neurogenesis in the striatum	Not discussed
Dey et al^20^	YAC128 mice	BM‐MSCs to overexpress either nerve growth factor (NGF) and/or BDNF	Green fluorescent protein mice	Increase in the number of region‐specific medium spiny neurons with increased expression of BDNF. The mice treated with MSCs overexpressing NGF and/or BDNF recovered from motor deficits	Not discussed
An et al^32^	R6/2	NSCs derived from gene corrected iPSCs	Lesch‐Nyhan syndrome carrier patients	The transplanted cells survived and differentiated into region specific DARPP 32 neurons	Not discussed
Fink et al^22^	R6/2 mice	Umbilical cord derived mesenchymal stem cells (UC‐MSCs)	E15 mouse pups	No recovery from motor deficits, however, improvement in spatial memory	Not discussed
Cicchetti et al^33^	HD patients	Fetal neuronal grafts	Healthy human tissue	The transplanted cells survived in the striatum, presence of mHTT transfer from the host cells to the graft cells in the striatum and cortex	Discussed—aggregates present
Rossignol et al^17^	R6/2 mice	BM‐MSCs	Wild‐type (WT) mice	HD mice treated with BM‐MSCs showed decreased motor deficits and sparing of spatial memory compared with the control mice. Increase in BDNF trophic factor was observed	Not discussed
Pollock et al^26^	YAC128 and R6/2 mice	BM‐MSCs genetically engineered to overexpress BDNF	Healthy human donors	Striatal atrophy was attenuated and histological analysis revealed an increase in neurogenesis. The R6/2‐treated mice showed increased life‐span compared with control mice	Not discussed
Jeon et al^34^	Healthy mice	iPSCs derived from HD skin fibroblasts	Juvenile HD patient	The mHTT aggregates translocated to the brain cells of the WT mice. HD‐like cognitive and motor‐related features observed in WT mice. Loss of medium spiny neurons in the striatum	Discussed—aggregates present
Al‐Gharaibeh et al^14^	YAC128 mice	Induced neural stem cells (iNSCs)	WT mice	The transplanted iNSCs differentiated into region‐specific medium spiny neurons. BDNF trophic factor increased. iNSCs‐treated animals showed lesser motor deficits compared with control mice	Not discussed
Reidling et al^17^	Q140 mice and R6/2 mice	Human embryonic stem cell‐derived neural stem cells (hNSCs)	Healthy human donors	Integration of the transplanted cells into the host circuit, reduction in mHTT aggregates with increased BDNF levels	Not discussed
Adil et al^15^	R6/2 mice	Medium spiny neurons derived from human pluripotent stem cells (hPSCs)	Mutated cardiomyocytes	Survival of cells and reduced motor deficits. The treated mice lived longer than the untreated mice	Discussed—aggregates present
Maxan et al^11^	HD patients	Fetal striatal stem cells	Healthy human donor	Survival of cells but no clinical improvement	Discussed—aggregates present
Masnata et al^35^	R6/2 pups	IPSC‐derived GABA neurons (iGABA) and human neuroblastoma cell line (SH‐SY5Y) incubated with mHTT fibrils	Human donors	The mHTT fibrils were transferred to the WT mice. Mice developed cognitive deficits and anxiety‐like behaviors 1 month after injection	Discussed—aggregates present
Yu Taeger et al^24^	R6/2 mice	BM‐MSCs	WT mice	Treated R6/2 mice had normal sleep cycle and increased survival. The deficit in the dopamine signaling cascade was rescued to some extent	Not discussed

Abbreviations: BDNF, brain‐derived neurotrophic factor; BM‐MSC, bone‐marrow derived mesenchymal stem cell; GFP, green fluorescent protein; HD, Huntington's disease; iPSC, induced pluripotent stem cell; mHTT, mutant form of the huntingtin protein; NGF, nerve growth factor; NSC, neural stem cell.

Given that unaltered autologous MSCs or iPSCs derived from the patient's own cells would still contain the HD gene, it is likely necessary to genetically alter these cells to remove or silence the mutated HD gene. As such, using genetic tools, for example, clustered regularly interspaced short palindromic repeats (CRISPR)‐Cas9 system, aimed at editing the CAG repeat expansion allele may prove beneficial and increase the clinical utility of stem‐cell‐based therapy.

## 5 HD GENE EDITING USING CRISPR‐CAS9

CRISPR, with its associated protein Cas9 nuclease, is a promising new gene editing tool that has been widely used for genome editing, including correcting various genetic disorders, such as HD. Reducing the number of CAG repeats from 40 or more to a normal length of less than 27 should be corrective, eliminating the toxic effects resulting from expression of the abnormal HTT protein. Since HD is autosomal dominant, one of the challenges is to achieve allele‐specific gene editing, sparing the normal allele, to produce a normal diploid genotype avoiding the adverse effects of silencing both alleles. Some researchers have identified specific protospacer adjacent motif (PAM) sequences that are present only at the site of the CAG repeat expansion in the mutant allele, which would allow specific targeting of the mutated allele without affecting the WT allele.^36^ That said, most studies to date have focused on the use of CRISPR‐Cas9 system for editing the HD gene, using either an allele‐specific or non‐allele‐specific strategy.

Since this is a concise review, only a few selected studies are described below. Shin and colleagues (2016) used single nucleotide polymorphism (SNP)‐based PAM sequence identification of the normal and the mutant allele to achieve allele‐specific HD gene editing in neural progenitor cells obtained from HD iPSCs. This study showed that the *mHTT* allele was specifically silenced, resulting in the production of only the WT protein.^36^

Similarly, Monteys and colleagues (2017) used SNPs (that create or destroy the PAM sequence) at the 5′ end of *HTT* exon 1 that are specific to the mutant HD allele to target and edit the *mHTT* gene using the CRISPR system. The study showed that following gene editing, mHTT expression was reduced by 40% in BACHD mice.^37^

In contrast, Yang and colleagues (2017) used the CRISPR‐Cas9 system to achieve non‐allele‐specific HD gene editing in HD140Q‐knockin mice. This study showed that there was an overall decrease in mHTT production, thereby reducing nuclear accumulation of aggregated mHTT. Moreover, reduction of mHTT resulted in fewer reactive astrocytes, decreasing inflammation in the HD brain. These HD mice showed improvement in motor deficits; however, overall life span was not increased compared with the controls.^38^

An in vitro study from our laboratory performed non‐allele‐specific gene editing in BM‐MSCs derived from YAC128 mice. The deletion mutations created following CRISPR‐Cas9‐based editing showed reduced CAG repeat expansions in the HD cells. Further analysis showed that mHTT was reduced compared with controls.^39^

Recently Xu and colleagues (2017) corrected the mutant allele in iPSCs derived from HD patients using CRISPR‐Cas9 technology. Following gene correction, the iPSCs expressed pluripotent markers which confirmed that these cells maintain their innate properties following gene correction. Furthermore, these cells were also able to differentiate into functional forebrain neurons and eventually showed reversal of the phenotypic abnormalities seen in the HD cells.^40^ This technology will allow transplantation of the patient's own cells following gene correction avoiding the need for immunosuppression.

A study in 2014 showed that HD patients who had received fetal neuronal grafts 10 years previously exhibited mHTT in the transplanted neuronal grafts in the striatum following tissue staining using EM48 or MW7 antibodies that stain mHTT. The mHTT aggregates were also found in the neurons, astrocytes, and microglia of cortex.^33^ Other researchers have transplanted HD skin fibroblasts (or iPSCs derived from them; with different CAG expansions) isolated from a juvenile HD patient into healthy adult CF‐1 strain mice. The results showed that the mice receiving the HD cells, irrespective of the number of CAG repeat expansions, showed motor and cognitive deficits that were similar to the cognitive and motor‐related features observed in HD patients. Furthermore, analysis of brain tissue showed that mHTT aggregates were also found in the host parenchyma, suggesting that the mHTT monomers or aggregates migrated from the graft tissue to the host tissue. Taken together, this study showed that mHTT can translocate from the transplanted cell grafts to the WT host tissue, which can lead to additional HD‐like pathology as well as loss of motor and cognitive ability.^34^ A study in 2019 showed prion‐like propagation of mHTT aggregates in vitro and in vivo. Incubation of iPSC‐derived GABA neurons, along with recombinant mHTT fibrils, showed that the human mHTT fibrils were taken up by the neurons altering their cell morphology, ultimately causing cell death. In addition, mHTT fibrils injected into the ventricles of adult WT mice led to cognitive deficits and anxiety‐like behaviors. Analysis of the tissue showed mHTT in the host brain 1 month following transplantation. However, the fibrils were undetectable approximately 14 months after transplantation, showing that the fibrils are eventually cleared.^35^

## 6 DO MHTT AGGREGATES TRANSFER TO TRANSPLANTED CELL GRAFTS IN A PRION‐LIKE FASHION?

Having discussed the successful therapeutic effects of different types of stem cell transplantation, use of autologous transplantation of *mHTT* gene corrected stem cells, as well as direct gene editing using CRISPR‐Cas9 system, a major issue that remains to be resolved was posed by the recent work of Cicchetti and colleagues. Their recent study indicates that mHTT aggregates exhibit “prion‐like” propagation from the host to the graft following transplantation of different types of stem cells into the HD brain.

An important consideration in assessing the applicability of stem cell transplantation for therapeutic purposes is the susceptibility of the transplanted cells to the mechanism(s) underlying the development of the disease being treated. Neurodegenerative diseases are associated with misfolded proteins that tend to form aggregates—the archetypal example being the prion diseases.^41^ The key feature in the development of a prion disease is the template‐assisted conversion of the normal cellular protein, PrP^C^, to the misfolded form, PrP^Sc^, linked to disease.^42^ Thus, prion diseases can be idiopathic, inherited through mutations in the prion protein gene, or acquired through infection. The ability to cause infection between organisms also appears to occur within an organism. Thus, the spread of a prion disease within the brain is not cell autonomous, but it rather spreads through infection from cell to cell. Familial forms of human prion disease are associated with mutations in the prion protein gene.^43^ In most cases, the mutant prion protein, following spontaneous conversion to the pathogenic form, is able to convert the normal prion protein. This has led to speculation that somatic mutation in one or a few cells may underlie the development of idiopathic disease.

While a number of other neurodegenerative diseases appear to spread in the brain through a similar mechanism, referred to as “prion‐like,” notably Alzheimer's disease (AD; tau) and Parkinson disease (PD; alpha‐synuclein^44^), not all demonstrate templating and only prion diseases have been shown to be transmissible. Of note, protein inclusions in neurons, suggestive of Lewy pathology, have been found in fetal tissue grafts characteristic of PD.^45^ Recent studies have suggested that spreading of host‐derived tau may result in pathology in grafted tissue in both PD and HD patients.^46^

In cells expressing mutant huntingtin exon 1, the protein spontaneously aggregates. In contrast, although pathogenic mutant prion protein associated with familial disease is misfolded during synthesis in cell models, the mutant prion protein does not achieve the pathological conformation. As an example, the T183A mutant prion protein is entirely retained in the endoplasmic reticulum due to its misfolding but is still proteinase‐K sensitive, pathology associated‐prion protein is proteinase‐K resistant, indicating that it has not converted to the pathogenic form.^47^ Although cell models show coincidence of aggregated mutant and normal huntingtin, mutant huntingtin does not appear to convert the normal huntingtin protein to a pathogenic conformation in patients. A study of allograft tissue recovered from HD patients after more than a decade showed extracellular huntingtin protein in the interstitial space of the normal graft tissue but not inside the cells.^33^

A further point of distinction between HD and other neurodegenerative diseases associated with protein misfolding is that the cytoplasmic protein aggregates appear to cause disease by globally interfering with proteostasis.^48^ In contrast, a comprehensive review of huntingtin biology by Saudou and Humbert (2016) provides compelling evidence for a dominant negative effect of mutant huntingtin on normal huntingtin function.^49^ With respect to HD, this may involve dysregulation of BDNF transport.^50^ In addition, huntingtin aggregates, formed after cleavage of the mutant huntingtin protein, are uniquely localized in the nucleus and have been reported to be either toxic^51^ or protective.^52^ In addition, the C‐terminal fragments generated by proteolysis are also toxic.^53^

## 7 CONCLUSIONS

Taken together, it is difficult to predict whether transplanted healthy or gene‐corrected stem cells would be subject to ongoing HD processes. Although neuronal tissue grafts showed evidence of extracellular huntingtin aggregates, the lack of intraneuronal aggregates may be due to the failure of the transplanted neurons to form synapses with the host neurons. Furthermore, if the stem cells only express normal huntingtin, they will not produce the proteolytic fragments generated by mutant huntingtin and may be spared. Exposing cells that express only normal huntingtin or cells that do not express huntingtin at all would provide answers to some of these questions. However, as suggested earlier, it may be that to achieve transfer of a sufficient quantity of mutant huntingtin to cells would require that they form synapses with cells expressing the mutant protein. In conclusion, the use of therapeutic stem cells for the treatment of HD remains a viable option. However, one has to consider the possibility of “prion‐like” transfer of mHTT aggregates following transplantation, especially when using gene‐corrected autotransplants.

## CONFLICT OF INTEREST

The authors declared no potential conflicts of interest.

## AUTHOR CONTRIBUTIONS

B.S.: manuscript preparation; R.B.P.: manuscript preparation, proof‐reading; G.L.D.: manuscript preparation, proof‐reading, approval of the manuscript; J.R.: conception, manuscript preparation, proof‐reading, approval of the manuscript.

## Data Availability

Data sharing is not applicable to this article as no new data were created or analyzed in this study.

## References

[1] Sugaya K , Vaidya M . Stem cell therapies for neurodegenerative diseases. Adv Exp Med Biol. 2018;1056:61‐84. 29754175 10.1007/978-3-319-74470-4_5

[2] Haddad MS , Wenceslau CV , Pompeia C , Kerkis I . Cell‐based technologies for Huntington's disease. Dement Neuropsychol. 2016;10:287‐295. 29213471 10.1590/s1980-5764-2016dn1004006 PMC5619267

[3] Havel LS , Wang C‐E , Wade B , Huang B , Li S , Li XJ . Preferential accumulation of N‐terminal mutant huntingtin in the nuclei of striatal neurons is regulated by phosphorylation. Hum Mol Genet. 2011;20:1424‐1437. 21245084 10.1093/hmg/ddr023 PMC3049362

[4] Thion MS , Humbert S . Cancer: from Wild‐type to mutant Huntingtin. J Huntington Dis. 2018;7:201‐208. 10.3233/JHD-180290 PMC6087435 29889077

[5] Labbadia J , Morimoto RI . Huntington's disease: underlying molecular mechanisms and emerging concepts. Trends Biochem Sci. 2013;38:378‐385. 23768628 10.1016/j.tibs.2013.05.003 PMC3955166

[6] Myers RH . Huntington's disease genetics. NeuroRx. 2004;1:255‐262. 15717026 10.1602/neurorx.1.2.255 PMC534940

[7] Rocha NP , Ribeiro FM , Furr‐Stimming E , et al. Neuroimmunology of Huntington's disease: revisiting evidence from human studies. Mediators Inflamm. 2016;2016:8653132. 27578922 10.1155/2016/8653132 PMC4992798

[8] Bachoud‐Lévi A , Bourdet C , Brugières P , et al. Safety and tolerability assessment of intrastriatal neural allografts in five patients with Huntington's disease. Exp Neurol. 2000;161:194‐202. 10683285 10.1006/exnr.1999.7239

[9] Hauser RA , Furtado S , Cimino CR , et al. Bilateral human fetal striatal transplantation in Huntington's disease. Neurology. 2002;58:687‐695. 11889229 10.1212/wnl.58.5.687

[10] Rosser AE , Barker RA , Harrower T , et al. Unilateral transplantation of human primary fetal tissue in four patients with Huntington's disease: NEST‐UKsafety report ISRCTN no 36485475. J Neurol Neurosurg Psychiatry. 2002;73:678‐685. 12438470 10.1136/jnnp.73.6.678 PMC1757375

[11] Maxan A , Mason S , Saint‐Pierre M , et al. Outcome of cell suspension allografts in a patient with Huntington's disease. Ann Neurol. 2018;84:950‐956. 30286516 10.1002/ana.25354 PMC6587549

[12] Takahashi K , Yamanaka S . Induction of pluripotent stem cells from mouse embryonic and adult fibroblast cultures by defined factors. Cell. 2006;126:663‐676. 16904174 10.1016/j.cell.2006.07.024

[13] Mu S , Wang J , Zhou G , et al. Transplantation of induced pluripotent stem cells improves functional recovery in Huntington's disease rat model. PLoS One. 2014;9:e101185. 25054283 10.1371/journal.pone.0101185 PMC4108311

[14] Al‐Gharaibeh A , Culver R , Stewart AN , et al. Induced pluripotent stem cell‐derived neural stem cell transplantations reduced behavioral deficits and ameliorated neuropathological changes in YAC128 mouse model of Huntington's disease. Front Neurosci. 2017;11:628. 29209158 10.3389/fnins.2017.00628 PMC5701605

[15] Adil MM , Gaj T , Rao AT , et al. hPSC‐derived striatal cells generated using a scalable 3D hydrogel promote recovery in a Huntington disease mouse model. Stem Cell Rep. 2018;10:1481‐1491. 10.1016/j.stemcr.2018.03.007 PMC5995679 29628395

[16] Joyce N , Annett G , Wirthlin L , Olson S , Bauer G , Nolta JA . Mesenchymal stem cells for the treatment of neurodegenerative disease. Regen Med. 2010;5:933‐946. 21082892 10.2217/rme.10.72 PMC3017479

[17] Rossignol J , Fink KD , Crane AT , et al. Reductions in behavioral deficits and neuropathology in the R6/2 mouse model of Huntington's disease following transplantation of bone‐marrow‐derived mesenchymal stem cells is dependent on passage number. Stem Cell Res Ther. 2015;6:9. 25971780 10.1186/scrt545 PMC4429666

[18] Rossignol J , Boyer C , Lévèque X , et al. Mesenchymal stem cell transplantation and DMEM administration in a 3NP rat model of Huntington's disease: morphological and behavioral outcomes. Behav Brain Res. 2011;217:369‐378. 21070819 10.1016/j.bbr.2010.11.006

[19] Kwan W , Magnusson A , Chou A , et al. Bone marrow transplantation confers modest benefits in mouse models of Huntington's disease. J Neurosci. 2012;32:133‐142. 22219276 10.1523/JNEUROSCI.4846-11.2012 PMC3571858

[20] Dey ND , Bombard MC , Roland BP , et al. Genetically engineered mesenchymal stem cells reduce behavioral deficits in the YAC 128 mouse model of Huntington's disease. Behav Brain Res. 2010;214:193‐200. 20493905 10.1016/j.bbr.2010.05.023

[21] Zuccato C , Marullo M , Vitali B , et al. Brain‐derived neurotrophic factor in patients with Huntington's disease. PLoS One. 2011;6:e22966. 21857974 10.1371/journal.pone.0022966 PMC3155522

[22] Fink KD , Rossignol J , Crane AT , et al. Transplantation of umbilical cord‐derived mesenchymal stem cells into the striata of R6/2 mice: behavioral and neuropathological analysis. Stem Cell Res Ther. 2013;4:130. 24456799 10.1186/scrt341 PMC3854759

[23] Ebrahimi MJ , Aliaghaei A , Boroujeni ME , et al. Human umbilical cord matrix stem cells reverse oxidative stress‐induced cell death and ameliorate motor function and striatal atrophy in rat model of Huntington disease. Neurotox Res. 2018;34:273‐284. 29520722 10.1007/s12640-018-9884-4

[24] Yu‐Taeger L , Stricker‐Shaver J , Arnold K , et al. Intranasal administration of mesenchymal stem cells ameliorates the abnormal dopamine transmission system and inflammatory reaction in the R6/2 mouse model of Huntington disease. Cell. 2019;8:595. 10.3390/cells8060595 PMC6628278 31208073

[25] Elbaz EM , Helmy HS , El‐Sahar AE , et al. Lercanidipine boosts the efficacy of mesenchymal stem cell therapy in 3‐NP‐induced Huntington's disease model rats via modulation of the calcium/calcineurin/NFATc4 and Wnt/β‐catenin signalling pathways. Neurochem Int. 2019;131:104548. 31539560 10.1016/j.neuint.2019.104548

[26] Pollock K , Dahlenburg H , Nelson H , et al. Human mesenchymal stem cells genetically engineered to overexpress brain‐derived neurotrophic factor improve outcomes in Huntington's disease mouse models. Mol Ther. 2016;24:965‐977. 26765769 10.1038/mt.2016.12 PMC4881765

[27] Snyder BR , Chiu AM , Prockop DJ , Chan AWS . Human multipotent stromal cells (MSCs) increase neurogenesis and decrease atrophy of the striatum in a transgenic mouse model for Huntington's disease. PLoS One. 2010;5:e9347. 20179764 10.1371/journal.pone.0009347 PMC2825266

[28] Lee S‐T , Chu K , Jung K‐H , et al. Slowed progression in models of Huntington disease by adipose stem cell transplantation. Ann Neurol. 2009;66:671‐681. 19938161 10.1002/ana.21788

[29] Reidling JC , Relaño‐Ginés A , Holley SM , et al. Human neural stem cell transplantation rescues functional deficits in R6/2 and Q140 Huntington's disease mice. Stem Cell Rep. 2018;10:58‐72. 10.1016/j.stemcr.2017.11.005 PMC5768890 29233555

[30] Ryu JK , Kim J , Cho SJ , et al. Proactive transplantation of human neural stem cells prevents degeneration of striatal neurons in a rat model of Huntington disease. Neurobiol Dis. 2004;16:68‐77. 15207263 10.1016/j.nbd.2004.01.016

[31] Lescaudron L , Unni D , Dunbar GL . Autologous adult bone marrow stem cell transplantation in an animal model of huntington's disease: behavioral and morphological outcomes. Int J Neurosci. 2003;113:945‐956. 12881187 10.1080/00207450390207759

[32] An MC , Zhang N , Scott G , et al. Genetic correction of Huntington's disease phenotypes in induced pluripotent stem cells. Cell Stem Cell. 2012;11:253‐263. 22748967 10.1016/j.stem.2012.04.026 PMC3608272

[33] Cicchetti F , Lacroix S , Cisbani G , et al. Mutant huntingtin is present in neuronal grafts in Huntington disease patients. Ann Neurol. 2014;76:31‐42. 24798518 10.1002/ana.24174

[34] Jeon I , Cicchetti F , Cisbani G , et al. Human‐to‐mouse prion‐like propagation of mutant huntingtin protein. Acta Neuropathol. 2016;132:577‐592. 27221146 10.1007/s00401-016-1582-9 PMC5023734

[35] Masnata M , Sciacca G , Maxan A , et al. Demonstration of prion‐like properties of mutant huntingtin fibrils in both in vitro and in vivo paradigms. Acta Neuropathol. 2019;137:981‐1001. 30788585 10.1007/s00401-019-01973-6 PMC6531424

[36] Shin JW , Kim K‐H , Chao MJ , et al. Permanent inactivation of Huntington's disease mutation by personalized allele‐specific CRISPR/Cas9. Hum Mol Genet. 2016;25:4566‐4576. 28172889 10.1093/hmg/ddw286 PMC6078600

[37] Monteys AM , Ebanks SA , Keiser MS , Davidson BL . CRISPR/Cas9 editing of the mutant Huntingtin allele in vitro and in vivo. Mol Ther. 2017;25:12‐23. 28129107 10.1016/j.ymthe.2016.11.010 PMC5363210

[38] Yang S , Chang R , Yang H , et al. CRISPR/Cas9‐mediated gene editing ameliorates neurotoxicity in mouse model of Huntington's disease. J Clin Invest. 2017;127:2719‐2724. 28628038 10.1172/JCI92087 PMC5490741

[39] Kolli N , Lu M , Maiti P , Rossignol J , Dunbar G . CRISPR‐Cas9 mediated Gene‐silencing of the mutant Huntingtin gene in an in vitro model of Huntington's disease. Int J Mol Sci. 2017;18:754. 10.3390/ijms18040754 PMC5412339 28368337

[40] Xu X , Tay Y , Sim B , et al. Reversal of phenotypic abnormalities by CRISPR/Cas9‐mediated gene correction in Huntington disease patient‐derived induced pluripotent stem cells. Stem Cell Rep. 2017;8:619‐633. 10.1016/j.stemcr.2017.01.022 PMC5355646 28238795

[41] Colby DW , Prusiner SB . Prions. Cold Spring Harb Perspect Biol. 2011;3:a006833. 21421910 10.1101/cshperspect.a006833 PMC3003464

[42] Vorberg IM . All the same? The secret life of prion strains within their target cells. Viruses. 2019;11:E334. 30970585 10.3390/v11040334 PMC6520713

[43] Prusiner SB . Biology and genetics of prions causing neurodegeneration. Annu Rev Genet. 2013;47:601‐623. 24274755 10.1146/annurev-genet-110711-155524 PMC4010318

[44] Vasili E , Dominguez‐Meijide A , Outeiro TF . Spreading of α‐Synuclein and tau: a systematic comparison of the mechanisms involved. Front Mol Neurosci. 2019;12:107. 31105524 10.3389/fnmol.2019.00107 PMC6494944

[45] Li J‐Y , Englund E , Holton JL , et al. Lewy bodies in grafted neurons in subjects with Parkinson's disease suggest host‐to‐graft disease propagation. Nat Med. 2008;14:501‐503. 18391963 10.1038/nm1746

[46] Cisbani G , Maxan A , Kordower JH , Planel E , Freeman TB , Cicchetti F . Presence of tau pathology within foetal neural allografts in patients with Huntington's and Parkinson's disease. Brain J Neurol. 2017;140:2982‐2992. 10.1093/brain/awx255 PMC5841208 29069396

[47] Capellari S , Zaidi SIA , Long AC , Kwon EE , Petersen RB . The Thr183Ala mutation, not the loss of the first glycosylation site, alters the physical properties of the prion protein. J Alzheimer Dis. 2000;2:27‐35. 10.3233/jad-2000-2104 12214108

[48] Trigo D , Nadais A , da Cruz E , Silva OAB . Unravelling protein aggregation as an ageing related process or a neuropathological response. Ageing Res Rev. 2019;51:67‐77. 30763619 10.1016/j.arr.2019.02.001

[49] Saudou F , Humbert S . The biology of Huntingtin. Neuron. 2016;89:910‐926. 26938440 10.1016/j.neuron.2016.02.003

[50] Drouet V , Ruiz M , Zala D , et al. Allele‐specific silencing of mutant huntingtin in rodent brain and human stem cells. PLoS One. 2014;9:e99341. 24926995 10.1371/journal.pone.0099341 PMC4057216

[51] Ross CA , Tabrizi SJ . Huntington's disease: from molecular pathogenesis to clinical treatment. Lancet Neurol. 2011;10:83‐98. 21163446 10.1016/S1474-4422(10)70245-3

[52] Miller JP , Holcomb J , Al‐Ramahi I , et al. Matrix metalloproteinases are modifiers of huntingtin proteolysis and toxicity in Huntington's disease. Neuron. 2010;67:199‐212. 20670829 10.1016/j.neuron.2010.06.021 PMC3098887

[53] El‐Daher M‐T , Hangen E , Bruyère J , et al. Huntingtin proteolysis releases non‐polyQ fragments that cause toxicity through dynamin 1 dysregulation. EMBO J. 2015;34:2255‐2271. 26165689 10.15252/embj.201490808 PMC4585462

